# Beneficial effect of prolonged heme oxygenase 1 activation in a rat model of chronic heart failure

**DOI:** 10.1242/dmm.011528

**Published:** 2013-04-16

**Authors:** Massimo Collino, Alessandro Pini, Niccolò Mugelli, Rosanna Mastroianni, Daniele Bani, Roberto Fantozzi, Laura Papucci, Marilena Fazi, Emanuela Masini

**Affiliations:** 1 Department of Drug Science and Technology, University of Turin, via P. Giuria 9, 10125 Turin, Italy; 2 Department of Preclinical and Clinical Pharmacology, University of Florence, Viale Pieraccini 6, 50139 Florence, Italy; 3 Department of Anatomy, Histology and Forensic Medicine, University of Florence, Viale Morgagni 85, 50134 Florence, Italy; 4 Department of Experimental Pathology and Oncology, University of Florence, Viale Morgagni 50, 50134 Florence, Italy; 5 Department of Physiopathology, Surgery Unit, University of Florence, Viale G. Pieraccini 6, 50139 Florence, Italy

## Abstract

We and others have previously demonstrated that heme oxygenase 1 (HO-1) induction by acute hemin administration exerts cardioprotective effects. Here, we developed a rat model of heart failure to investigate whether a long-term induction of HO-1 by chronic hemin administration exerted protective effects. Sprague Dawley rats that underwent permanent ligation of the left coronary artery were closely monitored for survival rate analysis and sacrificed on day 28 post-operation. Administration of hemin (4 mg/kg body weight) every other day for 4 weeks induced a massive increase in HO-1 expression and activity, as shown by the increased levels of the two main metabolic products of heme degradation, bilirubin and carbon monoxide (CO). These effects were associated with significant improvement in survival and reduced the extension of myocardial damage. The ischemic hearts of the hemin-treated animals displayed reduced oxidative stress and apoptosis in comparison with the non-treated rats, as shown by the decreased levels of lipid peroxidation, free-radical-induced DNA damage, caspase-3 activity and Bax expression. Besides, chronic HO-1 activation suppressed the elevated levels of myeloperoxidase (MPO) activity, interleukin 1β (IL-1β) production and tumor necrosis factor-α (TNFα) production that were evoked by the ischemic injury, and increased the plasma level of the anti-inflammatory cytokine IL-10. Interestingly, HO-1 inhibitor zinc protoporphyrin IX (ZnPP-IX; 1 mg/kg) lowered bilirubin and CO concentrations to control values, thus abolishing all the cardioprotective effects of hemin. In conclusion, the results demonstrate that chronic HO-1 activation by prolonged administration of hemin improves survival and exerts protective effects in a rat model of myocardial ischemia by exerting a potent antioxidant activity and disrupting multiple levels of the apoptotic and inflammatory cascade.

## INTRODUCTION

Gaseous transmitters are endogenous gases of small molecular weight that exert important physiological functions. Although carbon monoxide (CO) is most notably recognized for its toxicity, in recent years it has been shown to play an important role as an endogenous modulator of hemodynamic functions. Since the initial report in 1993 showing that CO serves as a signaling molecule (Verma et al., 1993), it has been implicated in a wide range of cellular responses and pathophysiological states that stretch well beyond the initial expectations. CO shares some of the physiological properties of nitric oxide (NO), including regulation of vascular tone in blood vessels and inhibition of platelet aggregation by elevating intracellular levels of cGMP. In addition, CO dilates blood vessels by directly activating calcium-dependent potassium channels (Wang et al., 1997). CO is generated during the process of heme degradation, which is catalyzed by heme oxygenase (HO), causing heme to be converted to CO, biliverdin and free iron (Maines, 1997). Biliverdin reduced to bilirubin acts as a potent antioxidant that protects cells against oxidative stress. Two isoforms of HO are expressed in the heart: HO-1, inducible, and HO-2, constitutive non-inducible (Peterson et al., 2009). HO participates in the homeostatic control of cardiovascular functions, including the regulation of blood pressure and the prevention of cardiac fibrosis (Wang et al., 2010). Heme has been reported to act as a promoter of low-density lipoprotein (LDL) oxidation, generating products that are toxic to endothelial cells, such as iron, thus suggesting a role of heme as a crucial risk factor in the development of atherogenesis and myocardial infarction (Grinshtein et al., 2003; Kumar and Bandyopadhyay, 2005). However, accumulation of hemin, the oxidized form of heme, in tissues such as the endothelium triggers heme degradation into bilirubin, iron and CO by inducing HO-1 expression and activity (Tsiftsoglou et al., 2006). In mice, cardiac-restricted HO-1 overexpression protects against ischemia and reperfusion injury, with improved contractile recovery and reduced infarct size (Yet et al., 2001). In contrast, transgenic mice that are heterozygous for targeted disruption of the *HO-1* gene, exhibit exaggerated cardiac injury and dysfunction after ischemia/reperfusion (I/R) (Yoshida et al., 2001). Very recently, increased serum levels of HO-1 have been shown to be associated with decreased severity of coronary artery diseases in individuals with acute myocardial infarction (Novo et al., 2011). Although we and others have previously demonstrated that myocardial HO-1 induction by acute treatment with the selective HO-1 inducer hemin protects against myocardial I/R injury (Masini et al., 2003; Giannini et al., 2005; Lakkisto et al., 2009; Yeh et al., 2009), the effects of chronic hemin administration in a model of prolonged cardiac ischemia have never been tested and it is not clear whether a long-term induction of HO-1 is beneficial or detrimental. Accordingly, this study was undertaken to extend the investigation of the effects of prolonged HO-1 activation by hemin in conditions associated with chronic myocardial ischemia.

**TRANSLATIONAL IMPACT**

**Clinical issue**

Although chronic heart failure is one of the leading causes of hospitalization, morbidity and mortality worldwide, effective pharmacological interventions are currently limited. Recent evidence suggests that induction of heme oxygenase 1 (HO-1), which is involved in the homeostatic control of cardiovascular function, by acute treatment with the selective HO-1 inducer hemin protects against myocardial ischemic injury. In line with this, it has been demonstrated that increased serum levels of HO-1 are associated with reductions in severity of coronary artery disease in individuals with acute myocardial infarction. However, the effects of chronic hemin administration and long-term induction of HO-1 in a model of prolonged cardiac ischemia have not yet been investigated.

**Results**

To determine the effects of long-term HO-1 induction, the authors developed a rat model of permanent ligation of the left coronary artery. This model demonstrates ventricular dysfunction and the typical features of cardiomyocyte injury related to severe oxygen starvation and dysfunction: severe myofibril hypercontraction and mitochondrial swelling. Administration of hemin (4 mg/kg body weight) every other day for 4 weeks induced a significant improvement in survival and reduced the extension of myocardial damage. Furthermore, the ischemic hearts of the hemin-treated animals displayed reduced local oxidative stress, apoptosis and inflammatory response compared with non-treated rats. These beneficial effects were associated with a massive increase in HO-1 expression and activity. The effects were abolished by pre-treatment with the selective HO-1 inhibitor zinc protoporphyrin IX (ZnPP-IX; 1 mg/kg), further validating the cardioprotective role of HO-1 activity.

**Implications and future directions**

This study demonstrates for the first time that selective and prolonged administration of agents that activate HO-1 could have a protective effect in conditions associated with chronic myocardial ischemic injury. The beneficial effects of HO-1 activation were shown to be due to a combination of anti-apoptotic and anti-inflammatory effects. This finding lends weight to the concept that increased expression and activity of HO-1 could be a newly identified defense mechanism against inflammation and oxidative stress factors that are present in the ischemic heart. Further studies are warranted to clarify the potential clinical relevance of these promising findings.

## RESULTS

### Effect of hemin on survival, infarct size and left ventricular function in rats exposed to myocardial ischemic injury

**Fig. 1.**
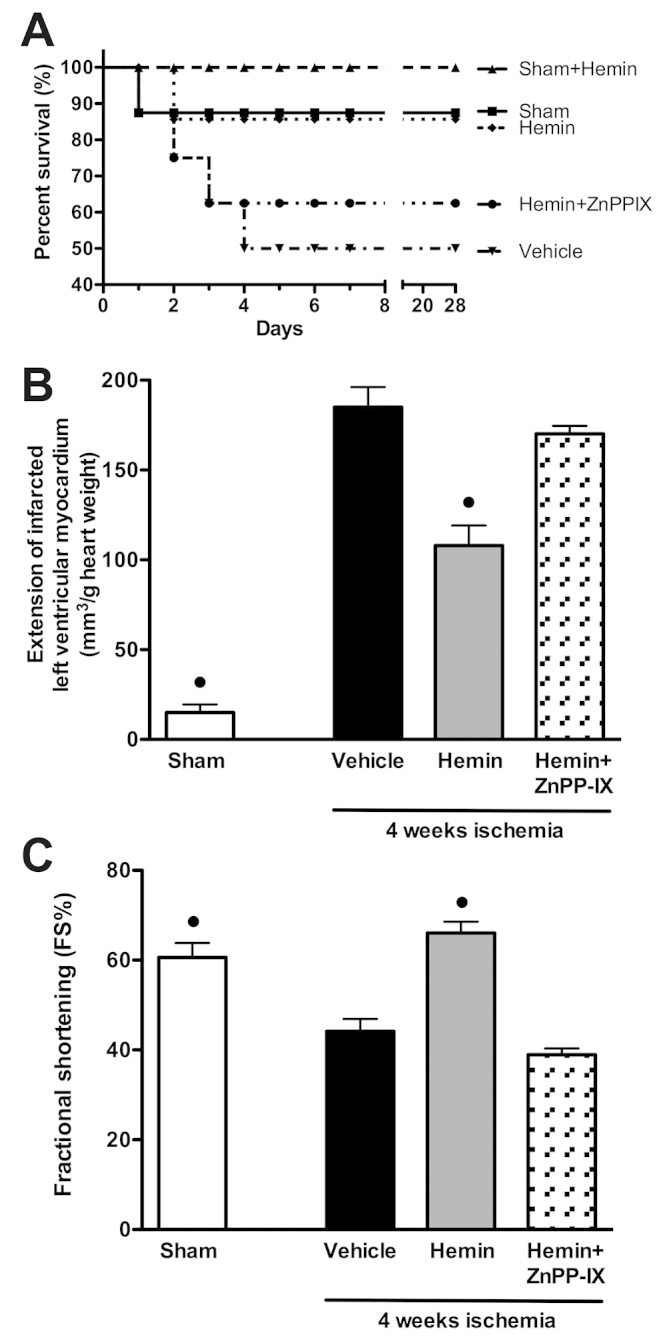
**Effects of hemin on survival, myocardial infarct size and cardiac function after permanent myocardial ischemia.** (A) 28-day Kaplan-Meier survival curve of mice subjected to LCA occlusion in the presence or absence of hemin (4 mg/kg body weight) and the HO-1 inhibitor ZnPP-IX (1 mg/kg body weight). (B) Effects of the HO-1 inducer hemin and the HO-1 inhibitor ZnPP-IX on the extension of infarcted LV myocardium with I/R-induced injury, evaluated on hearts stained with nitro-blue tetrazolium at 4 weeks of LCA occlusion. (C) Echocardiographic assessment of fractional shortening (FS%) 4 weeks following myocardial infarction in the presence or absence of hemin and the HO-1 inhibitor ZnPP-IX. Data are means ± s.e.m. of six animals/group. ^•^*P*<0.01 vs vehicle.

In total, 50% of rats that underwent regional myocardial ischemia and were treated with vehicle survived after 28 days. Chronic hemin administration significantly improved 28-day survival to 91.70% (Fig. 1A). The survival benefit of hemin was almost completely abolished in mice that were pretreated with the HO-1 inhibitor zinc protoporphyrin IX (ZnPP-IX) before hemin administration (Fig. 1A).

The results of the computer-assisted morphometry on ischemic hearts stained with nitroblue tetrazolium showed that administration of hemin every other day for 4 weeks induced a significant reduction in the extension of myocardial damage in comparison with that seen the untreated rats (Fig. 1B). As shown by echocardiography obtained at 4 weeks after left coronary artery (LCA) ligation (Fig. 1C), the percent of fractional shortening in the sham group was significantly higher than that in the ischemic group (60.58±7.21% and 44.17±5.11%, respectively), suggesting substantial ventricular dysfunction after 4 weeks of ischemia. In contrast, the cardiac functional recovery was significantly enhanced (66.04±5.61%) after hemin administration when compared with the ischemic group at the same period. Interestingly, the beneficial effects of hemin were almost abolished in rats that were pre-treated with the HO-1 inhibitor ZnPP-IX (Fig. 1C).

### Hemin reduces the cytological signs of ischemic injury

**Fig. 2.**
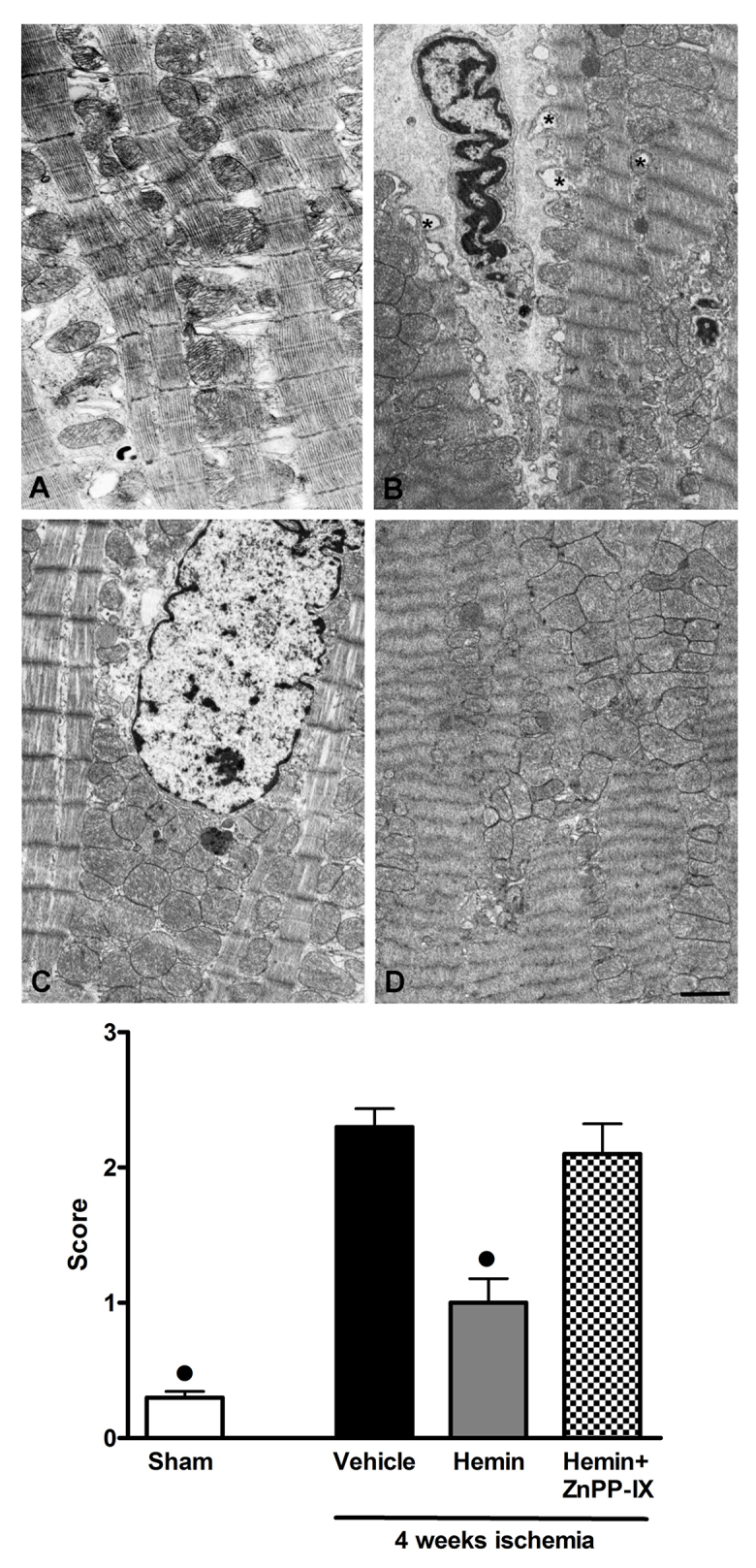
**Ultrastructural examination and quantitation of biopsies of LV tissues.** (A–D) Representative electron micrographs of cardiomyocytes from: sham-operated rats (A), showing normal myofibrils, mitochondria and organelle complement; rats undergoing LCA occlusion (B), showing severe myofibril hypercontraction, and mitochondrial swelling (asterisks); rats subjected to the ischemic insult and treated with 4 mg hemin/kg body weight (C), showing cytoplasmic edema, moderate myofibril hypercontraction and nearly normal microvessels; and rats subjected to the ischemic insult and treated with 4 mg hemin/kg body weight and the HO-1 inhibitor ZnPP-IX (1 mg/kg body weight) (D), showing severe myofibril hypercontraction. Scale bar: 1 μm. Lower panel: the quantification of ultrastructural tissue injury showed the beneficial effect of hemin treatment in reducing the signs of injury. Significance of differences (one-way ANOVA): ^•^*P*<0.01 vs vehicle.

When compared with the normal left ventricular (LV) morphology of sham-operated rats (Fig. 2A), the animals undergoing regional myocardial ischemia showed the typical features of cardiomyocyte injury related to severe oxygen starvation and dysfunction, namely severe myofibril hypercontraction and mitochondrial swelling (Fig. 2B). The LV myocardium from the rats subjected to the ischemic insult and treated with 4 mg hemin/kg body weight showed a marked reduction of the ultrastructural signs of injury, the most prominent changes being moderate myofibril hypercontraction and cytoplasmic edema (Fig. 2C). By contrast, rats subjected to myocardial ischemia and treated with 4 mg hemin/kg body weight together with the HO-1 inhibitor ZnPP-IX (1 mg/kg body weight) showed marked signs of ischemic injury, mainly consisting of severe myofibril hypercontraction (Fig. 2D). Quantification of ultrastructural tissue injury revealed that cell damage was significantly increased in the ischemic hearts as compared with the sham-operated ones and that hemin treatment robustly reduced the noted markers of cell injury. These beneficial effects were nullified by pre-treatment with the HO-1 inhibitor ZnPP-IX (Fig. 2, lower panel).

### Effects of hemin on the HO pathway

**Fig. 3.**
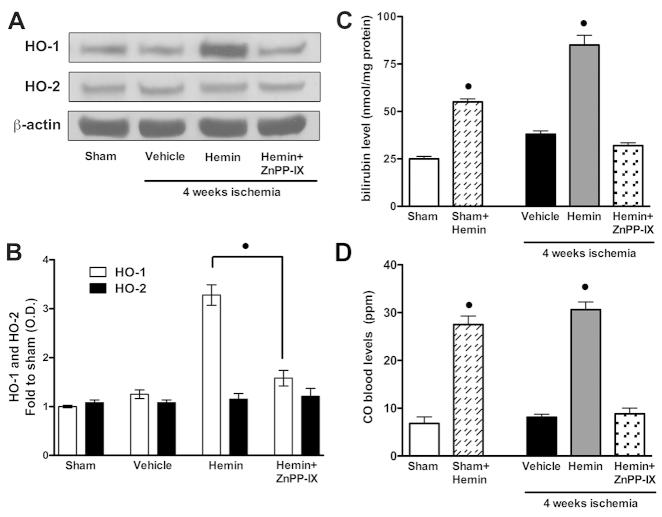
**Effects of hemin on the HO-CO pathway.** HO-1 and HO-2 expression levels (A) and related densitometric analysis (B), bilirubin production in heart homogenates (C) and CO blood values (D) were measured subsequent to sham operation (Sham) or chronic myocardial ischemia (vehicle). Rats were administered hemin or hemin+ZnPP-IX, as described in the Materials and Methods. Densitometric analysis of the related bands is expressed as relative optical density (OD) of the bands, corrected for the corresponding β-actin content and normalized using the related sham-operated band. Data are means ± s.e.m. of three separate experiments for western blot and five animals per group for bilirubin and CO detection. ^•^*P*<0.01 vs vehicle.

As shown in Fig. 3A and Fig. 3B, the ischemic insult did not affect either HO-1 or HO-2 protein expression in the rat heart, whereas hemin evoked a twofold increase in HO-1, but not HO-2, when administered to rats that underwent chronic ischemia. Hemin evoked not only HO-1 overexpression but also a massive increase in HO-1 activity, as shown by the increased blood levels of the two main metabolic products of heme degradation, bilirubin (85.7±8.3 nmol/mg protein; Fig. 3B) and CO (30.6±4.3 ppm; Fig. 3C), compared with measurements made in untreated ischemic animals (37.8±3.5 nmol/mg protein and 8.1±1.7 ppm, respectively). In rats that were pre-treated with the HO-1 inhibitor ZnPP-IX, the observed effects of hemin on HO-1 expression and activity were completely abolished.

### Effects of hemin on oxidative stress induced by ischemic injury

**Fig. 4.**
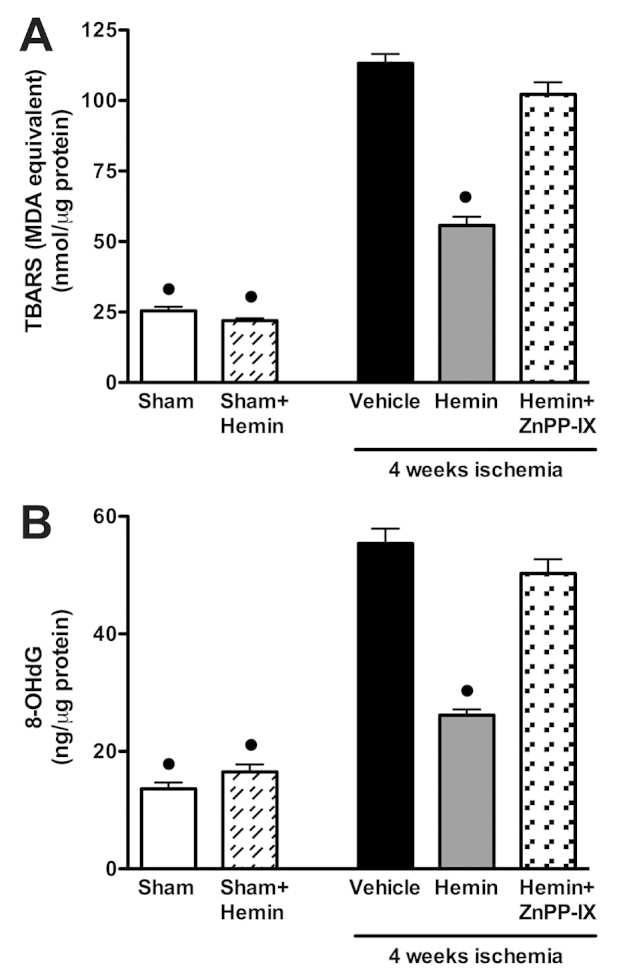
**Effects of hemin on lipid peroxidation and free-radical-induced DNA damage in LV tissue.** TBARS production (A) and 8-OHdG levels (B) were measured subsequent to sham operation (Sham) or myocardial ischemia in the absence (vehicle) or presence of hemin (4 mg/kg body weight) or hemin (4 mg/kg body weight) + ZnPP-IX (1 mg/kg body weight). Data are means ± s.e.m. of eight animals/group. ^•^*P*<0.01 vs vehicle.

Rats that had undergone permanent coronary occlusion exhibited a massive increase in tissue markers of oxidative stress, such as the production of thiobarbituric acid-reactive substances (TBARS; an index of peroxidation of cell membrane lipids) and 8-hydroxy-2′-deoxyguanosine (8-OHdG; a marker of free radical-induced DNA damage) (Fig. 4A,B, respectively). The robust increase in TBARS and 8-OHdG levels was blunted by administration of hemin for 4 weeks. The attenuation in oxidative stress levels obtained after hemin treatment was reversed by administration of the selective HO-1 inhibitor ZnPP-IX.

### Effects of hemin on leukocyte activation

**Fig. 5.**
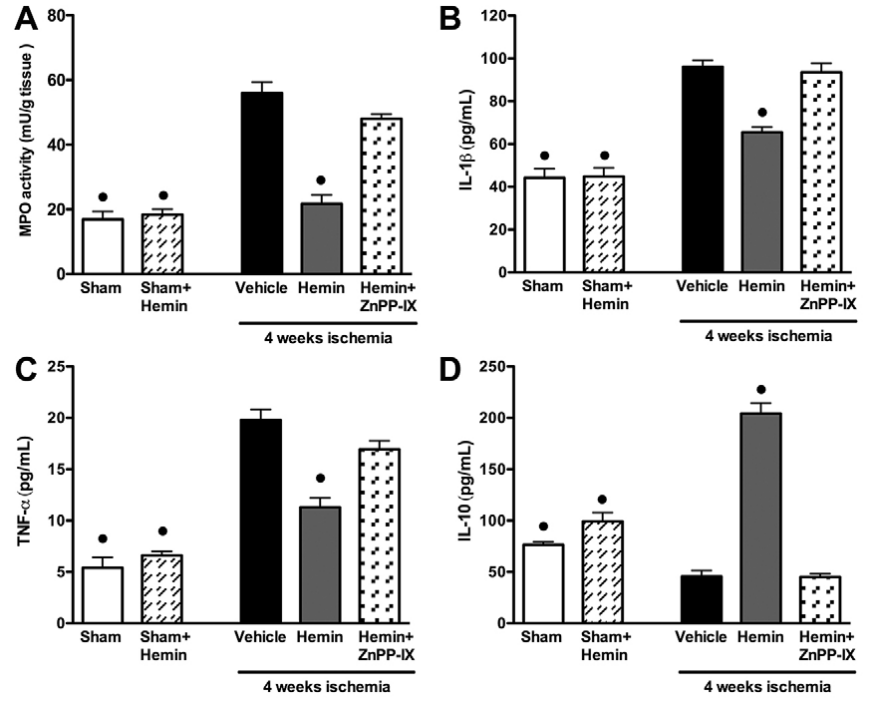
**Effects of hemin on leukocyte activation induced by regional chronic ischemia.** (A) MPO activity was measured in myocardial homogenates of sham-operated rats (sham) and rats that underwent 4 weeks ischemia (vehicle) in the presence or absence of hemin (4 mg/kg body weight) or hemin (4 mg/kg body weight) + ZnPP-IX (1 mg/kg body weight). IL-1β (B), TNFα (C) and IL-10 (D) levels were measured in the blood of rats exposed to 4 weeks of ischemic injury. Hemin (4 mg/kg body weight) or hemin (4 mg/kg body weight) + ZnPP-IX (1 mg/kg body weight) were administered by intraperitoneal injection every other day for 4 week, starting the same day as LCA occlusion. Data are means ± s.e.m. of eight animals/group. ^•^*P*<0.01 vs vehicle.

As shown in Fig. 5A, myocardial ischemia caused a robust increase in myeloperoxidase (MPO) activity, a specific marker of local neutrophil activity, in comparison with sham-operated rats (56.18±8.01 mU MPO/tissue g, 16.92±4.11 mU MPO/tissue g, respectively; *P*<0.05). Hemin administration almost completely abolished the ischemia-induced increase in MPO activity, whereas the HO-1 inhibitor ZnPP-IX abolished the hemin protective effects. A massive increase in the blood levels of IL-1β and TNFα, typical inflammatory cytokines released by activated leukocytes, was already detectable after 1 day of ischemia (190.94±11.05 pg/ml and 34.60±2.65 pg/ml, respectively), as compared with the sham-operated animals (44.26±9.52 pg/ml and 5.40±2.30 pg/ml, respectively), and it was still maintained at 28 days post-operation, although at lower levels (Fig. 5B,C, respectively). Administration of hemin prevented the rise in IL-1β and TNFα levels at day 1 after ischemia (99.20±11.80 pg/ml and 16.60±2.50 pg/ml, respectively), and similar results were obtained 28 days post-operation (Fig. 5B,C). Hemin administration also evoked a drastic increase in plasmatic content of IL-10, a well-known anti-inflammatory cytokine (Fig. 5D), whereas IL-10 levels were not significantly affected by ischemic injury. When rats were pre-treated with the selective HO-1 inhibitor ZnPP-IX, the protective actions of hemin on serum cytokine levels were consistently reduced.

### Effects of hemin on apoptosis markers

**Fig. 6.**
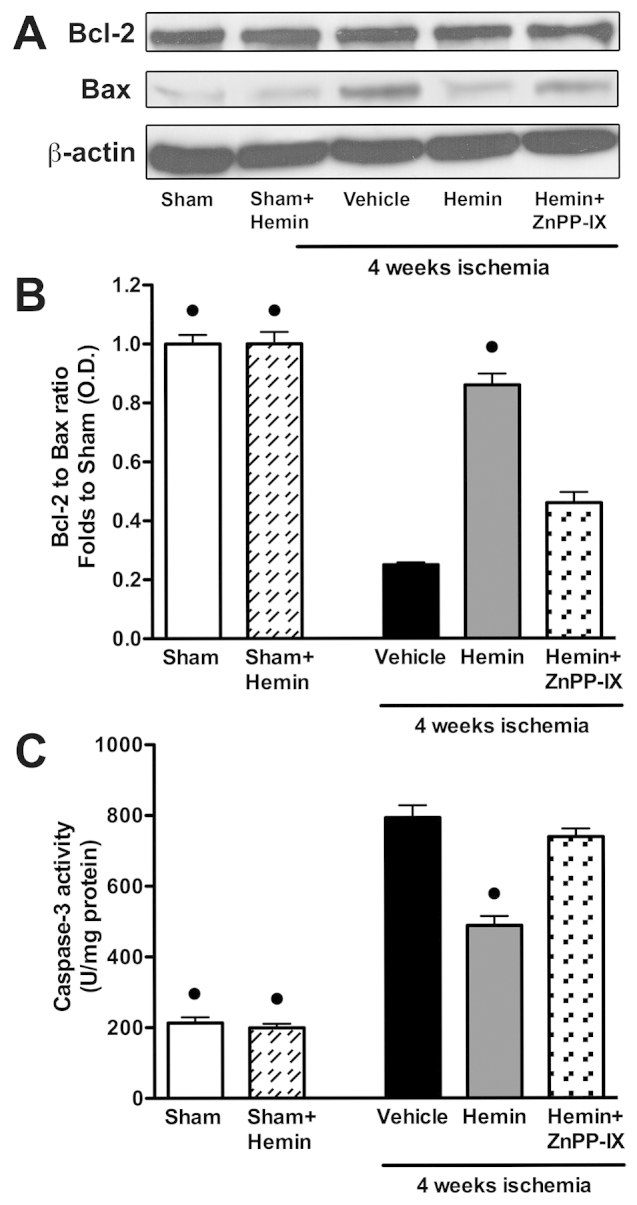
**Effects of hemin on markers of apoptosis in the hearts of rats that underwent myocardial ischemia.** (A,B) Representative western blot and corresponding densitometric analysis of the bands showing expression of Bcl-2 and Bax measured in the hearts of rats at 4 weeks after LCA occlusion in the presence or absence of hemin (4 mg/kg body weight) or hemin (4 mg/kg body weight) + ZnPP-IX (1 mg/kg body weight). Each immunoblot is from a single experiment and is representative of three separate experiments. (C) Caspase-3 activity was revealed by a fluorimetric assay in the hearts of sham-operated rats (sham) and rats that underwent 4 weeks of ischemia in the presence or absence of hemin (4 mg/kg body weight) or hemin (4 mg/kg body weight) + ZnPP-IX (1 mg/kg body weight). Data are means ± s.e.m. of four animals/group for caspase-3 activity and three separate experiments for western blot analysis. ^•^*P*<0.01 vs vehicle.

As shown in Fig. 6A, neither ischemia nor hemin administration modified the basal expression of Bcl-2 protein, a well-known suppressor of apoptosis. On the contrary, the levels of the mitochondrial proapoptotic protein Bax were significantly increased by the ischemic injury and this effect was abolished by exogenous hemin administration. Therefore, the Bcl-2:Bax ratio, an index of apoptosis signaling, was significantly reduced in animals subjected to myocardial ischemia in comparison with sham-operated rats and this effect was attenuated by treatment with hemin (Fig. 6B). When compared with sham-operated animals, there was a significant increase in caspase-3 activity within the ischemic myocardial tissue, which was significantly prevented by hemin administration (Fig. 6C). ZnPP-IX almost completely abolished the anti-apoptotic effects evoked by hemin administration.

## DISCUSSION

This study provides compelling evidence that chronic treatment with hemin, an HO-1 inducer, protects the rat heart from prolonged ischemic injury. The 4-week hemin regimen resulted in a significant reduction in infarct size and improvement in functional recovery, and these effects were associated with a significant increase in HO-1 myocardial expression. In contrast, we did not detect any change in HO-2 expression among different groups of animals and we demonstrated that the beneficial effects of hemin were abolished by the selective HO-1 inhibitor ZnPP-IX, thus supporting the notion that hemin cardioprotection was due to regulation of a subtype-selective expression of these isozymes. We also documented that induction of HO-1, following exposure to hemin, resulted in improved enzymatic activity, as shown by increased levels of the two main metabolites generated during the process of heme degradation, bilirubin and CO. We speculate that the release of heme catabolic products might act as an efferent signal to promote myocardial protection, as also suggested by a previous study demonstrating that the simultaneous administration of the two catabolic products of heme degradation reduces myocardial injury and improves cardiac function in the rat (Brennan et al., 2005). In our experimental model, the ability of the selective HO-1 inhibitor ZnPP-IX to lower bilirubin and CO concentrations to control values suggests that the increase in heme metabolite levels mostly depends on varied levels of HO-1 and further confirms the key role of HO-1 activation in hemin cardioprotection.

Although we and others have previously demonstrated that acute HO-1 upregulation occurs in response to ischemic injury in several organs, including the heart (Masini et al., 2003; Giannini et al., 2005; Lakkisto et al., 2009; Yeh et al., 2009), here we show for the first time that selective and prolonged pharmacological induction of HO-1 activity significantly ameliorates post-infarction myocardial remodeling. Recently, protective effects of chronic hemin administration have also been recorded in a rat model of hepatic ischemic injury (Fang et al., 2011). Similarly, chronic HO-1 induction has been reported to modulate renal hemodynamics and renal excretory function in ischemic kidney diseases (Ferenbach et al., 2010). In fact, although renal deposit of heme proteins can induce tubular injury and, thus, contribute to decreased kidney function, HO-1 has been demonstrated to be rapidly upregulated in the kidney exposed to heme proteins (Nath and Katusic, 2012). Such autoregulatory HO-1 induction represents a unique countervailing response that mitigates the sensitivity to organ injury. Although the products of HO-1 are capable of activating an array of downstream cytoprotective responses, the mechanisms underlying these remain poorly understood and we are still missing a unified theory to explain the protective effects of hemin. Initially, the protective role of HO-1 induction against ischemic injury has been related to the potent antioxidant activity of the products of the HO system. A large-scale prospective study of British men revealed that subjects in the midrange of serum bilirubin concentration had the lowest incidence of ischemic heart disease relative to those subjects displaying the lowest fifth of serum bilirubin distribution (Breimer et al., 1994).

High serum bilirubin levels have also been associated with a decreased risk for the development of coronary artery disease and atherosclerosis in humans (Mayer, 2000). In keeping with these previous reports, here we show that hemin administration evokes bilirubin production and increased CO blood levels with a successive remarkable decrease in tissue markers of oxidative stress and free-radical-induced DNA damage. Recent findings have led to a re-definition of the HO-1 pathway as not only an anti-oxidative mechanism but also a more complex and better coordinated cytoprotective system, with effects on several inflammatory and apoptotic signal transduction pathways (Abraham and Kappas, 2005). Accordingly, in the present study we found that hemin-induced improved outcome is accompanied by reductions in leukocyte activation as well as cardiomyocyte apoptosis and these beneficial effects are almost completely reversed by the administration of the selective HO-1 inhibitor ZnPP-IX, thus further confirming the key role of HO-1 induction in myocardial protection. Chronic hemin administration almost suppresses the elevated levels of MPO activity and IL-1β and TNFα production evoked by the ischemic injury and increases the plasma level of the anti-inflammatory cytokine IL-10, which suggests a shift from a balanced cytokine response to an anti-inflammatory state.

The suppression of inflammatory cytokines by hemin can be explained, at least in part, by hemin-induced CO overproduction, because CO has been previously proved to exert potent anti-inflammatory effects at low concentrations. In a murine model, CO preconditioning had resulted in the reduction of serum TNFα, IL-1β and IL-6 (Otterbein et al., 2000; Morse et al., 2003). As recently reported, the main mechanisms whereby CO affects the inflammatory response evoked by the ischemic injury include the downregulation of Toll-like receptor trafficking and activation (Wang et al., 2009), direct activation of the p38 mitogen-activated protein kinase (Otterbein et al., 2000), and the increased expression of the 70-kDa heat-shock protein (Kim et al., 2005). In addition, HO-1 activation has been demonstrated to downregulate the inflammatory response by blocking the release of NO from inducible NO synthase (iNOS) and the expression of granulocyte-macrophage colony-stimulating factor (Zuckerbraun et al., 2005). Thus, it is possible that production of CO, as a result of increased expression and activity of HO-1, could be a newly identified defense mechanism against inflammation and other related stress factors present in the ischemic heart.

Complementary to the attenuation in the inflammatory response, hemin further reduces myocardial apoptosis. Apoptosis with an increase in the activation of caspase-3 has been reported in cardiomyocytes of patients undergoing transplantation (Narula et al., 1999). Cardiac-specific overexpression of the anti-apoptotic regulator Bcl-2 substantially reduces infarct size, cardiac myocyte apoptosis and cardiac dysfunction following myocardial infarction (Chen et al., 2001). Similarly, mice lacking the multidomain proapoptotic Bax demonstrate reductions in myocardial infarct size and dysfunction. The cellular stoichiometry of Bcl-2 family members compared with their pro-apoptotic homolog, Bax, clearly defines the vulnerability of the cell to most death stimuli. In our study, hemin administration reduced the increase in pro-apoptotic Bax levels induced by the ischemic insult and thereby reversed the Bcl-2:Bax ratio. Besides, hemin administration prevented caspase-3 activation, thus providing a further crucial lead in defining hemin anti-apoptotic properties. Our findings are in keeping with the study of Tang and colleagues showing that a plasmid-mediated HO-1 gene-transfer strategy can provide cardiac-specific protection from ischemic injury by reducing apoptotic pathways (Tang et al., 2004). A crucial role in mediating hemin anti-apoptotic effects seems to be exerted by CO, whose anti-apoptotic potential was first demonstrated in different cell cultures, including mouse fibroblasts (Petrache et al., 2000) and endothelial cells (Brouard et al., 2000). The anti-apoptotic effect of CO involved activation of the p38 MAPK isoform, and inhibition of Fas and/or FasL expression and other apoptosis-related factors, including caspase-3, Bax proteins and mitochondrial cytochrome *c* release (Zhang et al., 2003). An *in vitro* study has recently demonstrated that preconditioning of Kit-positive human cardiac stem cells (hCSCs) with an HO-1 inducer promotes cellular resistance to oxidative stress and apoptosis through activation of the MAPK signaling pathway and release of various cytokines (Cai et al., 2012).

In conclusion, our results show for the first time that, when administered every other day for 4 weeks, hemin induces a significant reduction in myocardial infarct size in the rat. The beneficial effect of chronic hemin administration is associated with the prolonged activation of the HO-1 pathway, which leads to inhibition of the excessive inflammatory response as well as local apoptotic activation. This modulation by hemin of both inflammatory and apoptotic events well exemplifies accumulating evidence emphasizing the complexity of the molecular and cellular regulation of the pathophysiology of myocardial ischemia and, most notably, suggests that the administration of agents that activate HO-1 might be of benefit in conditions associated with ischemic injury. However, further studies are warranted to clarify the potential clinical relevance of our findings.

## MATERIALS AND METHODS

### Animals

A total of 60 male albino rats, Sprague Dawley strain, weighing 250–300 g (Morini, Reggio Emilia, Italy) were quarantined for 7 days with laboratory chow (Rodentia, Bergamo, Italy) and water *ad libitum*. The experimental protocol was designed in compliance with the EC Directive 86/609/EEC for animal experiments of the European Parliament and was approved by the Animal Care Committee of the University of Florence (Italy).

### Surgical procedure and treatments

The rats were anesthetized with intramuscular (i.m.) injection of a mixture of droperidol (5 mg/kg body weight) and fentanyl (0.20 mg/kg body weight), supplemented as needed, and monitored for body temperature, respiration pattern, loss of righting reflex, unresponsiveness and corneal reflexes. A cannula was inserted into the trachea and the animals were ventilated with air using a Palmer pump (Ugo Basile, Comerio, Italy). Subcutaneous peripheral limb electrodes were inserted and an electrocardiogram (ECG) was continuously recorded for the entire duration of the experiment. All rats underwent thoracotomy at the fifth left intercostal space, the pericardium was opened and a loose 00 braided silk suture was placed around the left anterior descending coronary artery. The chest was then closed with a silk suture to minimize heart displacement. Rats were allowed to equilibrate for 20 minutes to enable ECG values to stabilize. Permanent ligation of the LCA was induced by tightening the threads of the coronary suture. Animals were awakened with an intravenous injection of naloxone (0.4 mg/kg) and kept for at least 3 hours in a 37°C incubator to ensure that postoperative recovery was satisfactory. Tail vein blood was collected at 24 hours after reperfusion for cytokine analysis and plasma was stored at −80°C until time of assay, after centrifugation for 10 minutes at 8200 ***g***. Animals were randomly allocated into five different groups (*n*=12 per group): the sham group (rats undergoing the same surgical procedures as above but without the tightening of the coronary sutures); sham hemin group; vehicle ischemic group; ischemic hemin group; and ischemic hemin+ZnPP-IX group. The vehicle ischemic group was injected with normal saline intraperitoneally every other day for 4 weeks. Hemin (4 mg/kg body weight) was administered by intraperitoneal (i.p.) injection every other day for 4 weeks, starting the same day as LCA occlusion. The hemin+ZnPP-IX group of rats was pre-treated with ZnPP-IX (1 mg/kg body weight i.p.) 12 hours before hemin administration. The doses of hemin and the potent HO-1 inhibitor ZnPP-IX (*K*_i_=3 nM) were chosen based on those previously shown to protect from ischemic injury in the rat (Masini et al., 2003; Giannini et al., 2005). Animals were monitored for 28 days for all-cause mortality and a Kaplan-Meier 10-day survival curve was generated.

### Echocardiographic assessment of LV function

Transthoracic echocardiography using a 12-MHz linear array transducer was performed 1 week before LCA ischemia to avoid any anesthetic effects using a high-resolution ultrasound system. At 4 weeks following the myocardial infarction (post myocardial infarction), echocardiography images were obtained and analyzed once again. The rats (*n*=5 in each group) were anesthetized by 3% isoflurane; loss of righting reflex, unresponsiveness and corneal reflexes were used to check for successful anesthesia. Rats were placed in a supine position and their chests were shaved. LV internal chamber dimension in end-diastole (LVDd) and in end-systole (LVDs) were measured in each heart. LV fractional shortening (FS%) was calculated as (LVDd−LVDs)/LVDd×100%.

### Analysis of CO blood levels

Double samples of arterial blood were collected in gas-tight tubes and kept at 4°C until CO analysis, which was performed using an ultra-trace level gas detection system (RGA3 Reduction Gas Analyzer, SAES Getters, Milan, Italy), as described elsewhere (Tarquini et al., 2009).

### Determination of infarct size

At 4 weeks after LCA occlusion, the rats were anesthetized with 3% isoflurane, and 2 ml Evans Blue (Sigma Co, St Louis, MO) were retrogradely injected with a thin catheter inserted into the carotid artery to delineate the *in vivo* area at risk (AAR) (Masini et al., 2002). To distinguish between viable ischemic and infarcted tissue, the pnitro blue tetrazolium (NBT) dye exclusion method was used. The animals were killed by cervical dislocation. Upon removal, the hearts were attached to a Langendorff apparatus through a cannula introduced into the aorta and perfused with 10 ml of 1% NBT dissolved in a modified Tyrode solution, at a constant pressure of 40 cm of water at 37°C for 20 minutes. The hearts were detached from the cannula, weighed, fixed in buffered 4% formaldehyde for 12 hours, and the ventricles sectioned in 1-mm transverse slices from the apex to the ligature. In each slice, the bound areas of the unstained area on the upside surface were traced onto a superimposed acetate sheet and the encircled area was measured by computer-assisted morphometry. The LV area, AAR, the area of infarction of each slide and the total volume of the damaged myocardium were then determined as previously described (Masini et al., 2002). All measurements and calculations were performed by two individuals (A.P. and R.M.), who were blinded to treatment status.

### Ultrastructural examination

Electron microscopic examination was carried out on ultrathin sections of heart tissue fragments stained with uranyl acetate and alkaline bismuth subnitrate, and examined under a JEM 1010 electron microscope (Jeol, Tokyo, Japan) at 80 Kv. In each fragment, two series of six to eight ultrathin sections cut at two different levels (each series put on an electron microscopy grid) were examined and photographed. Myocyte injury was quantitated from electron micrographs (final magnifications ranging from 3000× to 20,000×) using a previously described method (Bani et al., 1998). The criteria used are reported in Table 1. Each animal was assigned a score for myocyte injury and the average values (mean ± s.e.m.) of each group were then calculated.

**Table 1.**
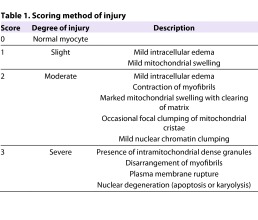
Scoring method of injury

### Determination of HO activity

Cardiac samples were homogenized and incubated for 30 minutes at 37°C with 50 μl of rat liver biliverdin reductase to convert biliverdin to bilirubin (Llesuy and Tomaro, 1994). The level of bilirubin was measured spectrophotometrically using a Sigma diagnostic procedure (Sigma, St Louis, MO) as previously reported (Ndisang et al., 2001).

### Evaluation of MPO activity

MPO activity, which can be used as a marker for neutrophil accumulation in tissues, was determined as previously described (Bani et al., 1998).

### Determination of TBARS

TBARS are end products of cell membrane lipid peroxidation used as a reliable marker of myocardial cell damage. TBARS were determined by measurement of the chromogen obtained from the reaction of MDA with 2-thiobarbituric acid according to Aruoma et al. (Aruoma et al., 1989).

### Determination of 8-OHdG

DNA isolation from cardiac tissue homogenates was performed according to Masini et al. (Masini et al., 2005). Samples of DNA extract were used for 8-OHdG determination using a Bioxytech enzyme immunoassay kit (Oxis, Portland, OR), following the instructions provided by the manufacturer. The values are expressed as ng of 8-OHdG per μg proteins.

### Measurement of caspase-3 activity

The enzymatic activity of caspase-3 was determined using the Ac-Asp-Glu-Val-Asp-AMC (Ac-DEVD-AMC; Bachem) fluorescent substrate (Stennicke and Salvesen, 1997). Substrate cleavage was monitored fluorimetrically (Spectrofluo JY3 D, Jobin Yvon, Paris, France) at 380-nm excitation and 460-nm emission wavelengths. Data are expressed as arbitrary units/mg proteins. One unit of enzyme activity is defined as the amount of enzyme required to liberate 40 μmol of Ac-DEVD-AMC over 60 minutes at 37°C.

### Determination of TNFα, IL-1β and IL-10 production

Cytokines were measured using commercial enzyme-linked immunosorbent assay (ELISA) kits (Cayman Chemical, Ann Arbor, MI), following the protocol provided by the manufacturer, and results are expressed as pg/ml.

### Western blot analysis

Western blots were carried out as previously described (Collino et al., 2008). Proteins were separated by 10% sodium dodecyl sulphate (SDS)-PAGE and transferred to polyvinyldenedifluoride membranes. The membranes were then incubated with primary and then secondary antibodies and developed using the ECL detection system. The immunoreactive bands were visualized by autoradiography and the density of the bands was evaluated densitometrically using Gel Pro^®^Analyser 4.5, 2000 software (Media Cybernetics, Silver Spring, MD).

### Statistical analysis

The reported data are expressed as mean ± s.e.m. Statistical analysis was performed by either one-way analysis of variance (ANOVA) test followed by Student-Newman-Keuls multiple comparison test or by Student’s *t*-test for unpaired values. Calculations were carried out using a GraphPad Prism 2.0 statistical program (GraphPad Software, San Diego, CA). *P*<0.05 was considered significant.

### Materials

Unless stated otherwise, all compounds were purchased from the Sigma-Aldrich Company Ltd (St Louis, MO). Antibodies against HO-1, HO-2, Bcl-2 and Bax were from Santa Cruz Biotechnology (Santa Cruz, CA). Luminol ECL detection reagents were from Amersham (Buckinghamshire, UK).
